# Supplementary material for: Cell-autonomous restoration of splicing homeostasis and RP11 phenotype in patient-derived RPE and retinal organoids by PRPF31.AAV gene therapy
Source: Nat Commun. 2026 Sep 30;17:10365. doi: 10.1038/s41467-026-78065-z (PMC13627695; doi:10.1038/s41467-026-78065-z)
Supplement: Supplementary file 1 [file 41467_2026_78065_MOESM1_ESM.pdf]

Cell-autonomous restoration of splicing homeostasis and RP11 phenotype in patient-derived RPE and retinal organoids by *PRPF31*.AAV gene therapy

Maria Elia [1], Valda Pauzuolyte [2], Maria Georgiou [1], Mark Basche [2], Carina Hansohn [3,4], Elton Vasconcelos [5], Robert Atkinson [1], Sushma Grellscheid [6], Colin A Johnson [7], Gerrit Hilgen [8], Henning Urlaub [3,4], Alexander J. Smith [2], Sina Mozaffari-Jovin [3#], Robin R. Ali [2#] and Majlinda Lako [1#]

# These authors jointly supervised this work

Email: majlinda.lako@ncl.ac.uk

1. Biosciences Institute, Faculty of Medical Sciences, Newcastle University, Newcastle upon Tyne, UK.
2. Centre for Gene Therapy & Regenerative Medicine, Faculty of Life Sciences & Medicine, King's College London, London, UK
3. Department of Clinical Chemistry, University Medical Center Göttingen, Göttingen, Germany
4. Max Planck Institute for Multidisciplinary Sciences, Göttingen, Germany
5. Leeds Omics, University of Leeds, Leeds, UK
6. Department of Informatics, University of Bergen, Bergen, Norway
7. Leeds Institute of Medical Research, Faculty of Medicine & Health, University of Leeds, Leeds, UK
8. School of Geography and Natural Sciences, Northumbria University, Newcastle upon Tyne, UK

Keywords: PRPF31, retinal organoids, gene therapy, retinal pigment epithelium cells, cilia, splicing, retinitis pigmentosa.

Supplementary information:

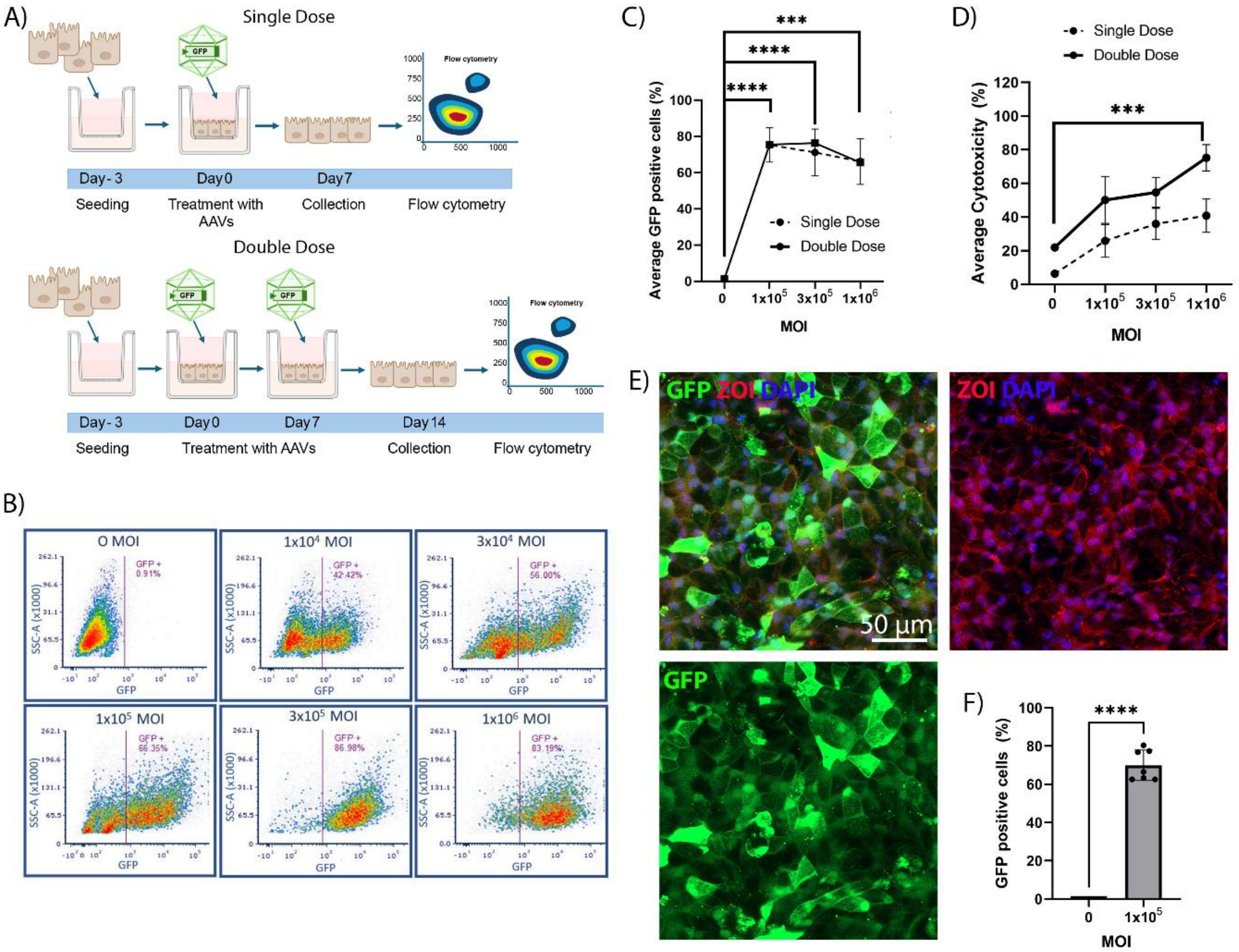

Figure S1: Optimisation of ShH10.AAV Transduction Efficiency and Safety in iPSC-Derived RPE Cells.

(A) Experimental workflow. Schematic representation of the transduction protocol for iPSC-derived RPE cells utilising single or double dosing regimens of ShH10.CMV.GFP.

(B) Single dose-response analysis via flow cytometry. Representative scatter plots and population gating illustrate the percentage of GFP-positive (GFP+) RPE cells across a MOI titration range of 0 to $1{\times}10^6$ vg/cell.

(C) Transduction efficiency of single vs. double dosing. Comparative analysis of GFP+ RPE cells following a single dose (dotted line) or double dose (continuous line) of ShH10.CMV.GFP.

(D) Cytotoxicity profiling. Evaluation of cellular toxicity (%) across the three most efficient multiplicities of infection (MOIs) for both single and double dosing regimens. A significant increase in cytotoxicity was observed at the highest MOI in the double-dose group.

(E) Visualisation of viral transgene expression following single dosing with ShH10.CMV.GFP AAVs. Immunofluorescence microscopy confirms robust GFP expression (green) in RP11-RPE cells transduced with ShH10.CMV.GFP at an MOI of $1{\times}10^5$ (left) compared to untreated controls (right). Nuclei are counterstained with DAPI (blue). Scale bar: 20 µm.

(F) Quantitative validation of GFP expression. Image-based quantification confirms a significant increase in GFP+ cells following treatment with MOI of 1×10^5 vg/cell ShH10.CMV.GFP AAVs. Data are presented as mean ± SEM (n=3).

Statistical Analysis: Data in (C) and (D) were analysed using a two-way ANOVA followed by Šídák's multiple comparisons test (n=5). Statistical significance is indicated as follows: ***p<0.001, ****p<0.0001. Data in (F) were analysed using a two-sided unpaired t-test (n=5). Source data are provided as a Source Data file.

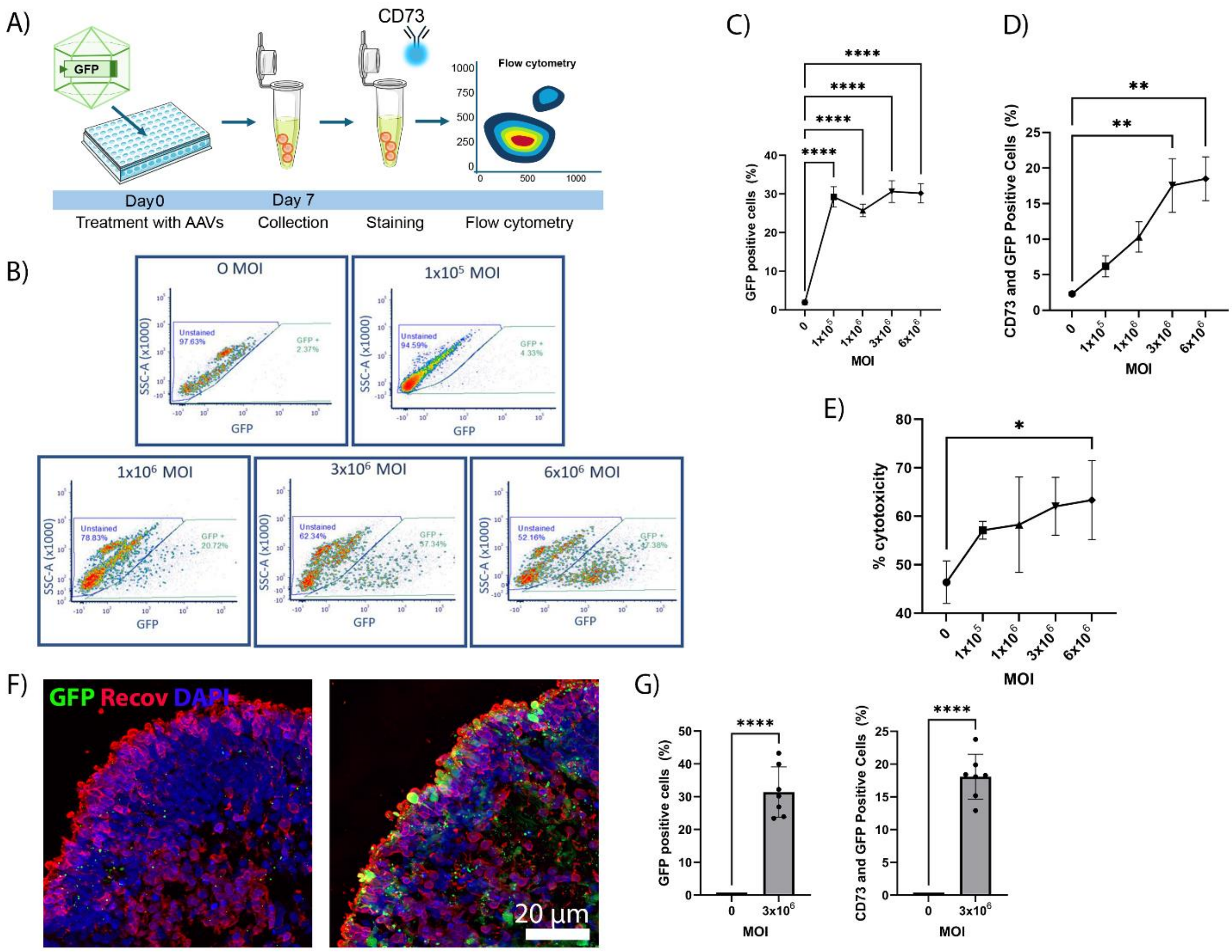

Figure S2: Assessment of ShH10.AAV Transduction Efficiency and Photoreceptor Tropism in iPSC-derived ROs.

(A) Experimental workflow for organoid transduction. Schematic of the protocol for treating day 150 (D150) iPSC-derived ROs with ShH10.CMV.GFP AAVs. To quantify efficiency, ROs were dissociated into single-cell suspensions and immunostained with the photoreceptor marker CD73 before flow cytometric analysis. Illustrations adapted from Servier Medical Art (https://smart.servier.com), licensed under CC BY 4.0 (https://creativecommons.org/licenses/by/4.0/).

(B) Representative flow cytometry profiles. Gating strategy and fluorescence plots for ROs transduced with ShH10.CMV.GFP AAVs across a MOI range of 0-6x10^6 vg/cell.

(C) Global transduction efficiency. Comparison of the percentage of GFP% cells in control and RP11-ROs across four MOIs. Statistical significance was determined via ordinary one-way ANOVA with Dunnett's multiple comparisons test.

(D) Targeted photoreceptor transduction. Percentage of CD73+/GFP+ dual-positive cells, confirming ShH10 tropism for the photoreceptors residing in the apical aspect of ROs. Statistical significance was assessed by ordinary one-way ANOVA.

(E) Dosage-dependent cytotoxicity. Evaluation of LDH release/cytotoxicity (%) in ROs. Significant toxicity was observed only at the highest MOI ($6x10^6$ vg/cell). Data were analysed using two-way ANOVA with Dunnett's multiple comparisons test (*$p < 0.1$).

(F) Histological validation of transgene expression. Immunofluorescence imaging of RO sections confirms high GFP expression (green) localised to the photoreceptor layer in transduced ROs ($3x 10^6$ vg/cell, right panel) compared to untreated controls (left panel). Nuclei are counterstained with DAPI (blue). Scale bar: 20 μm.

(G) Quantitative image analysis. Volumetric quantification of GFP fluorescence confirms a significant increase in signal in ROs treated with 3×10^6 vg/cell compared to controls. Data analysed via unpaired t-test.

Statistical Analysis: All data are presented as mean ± SD, n = 3, for (G) n = 7 (*$p < 0.05$, **$p < 0.01$, ****$p < 0.0001$). Source Data are provided as a Source Data file.

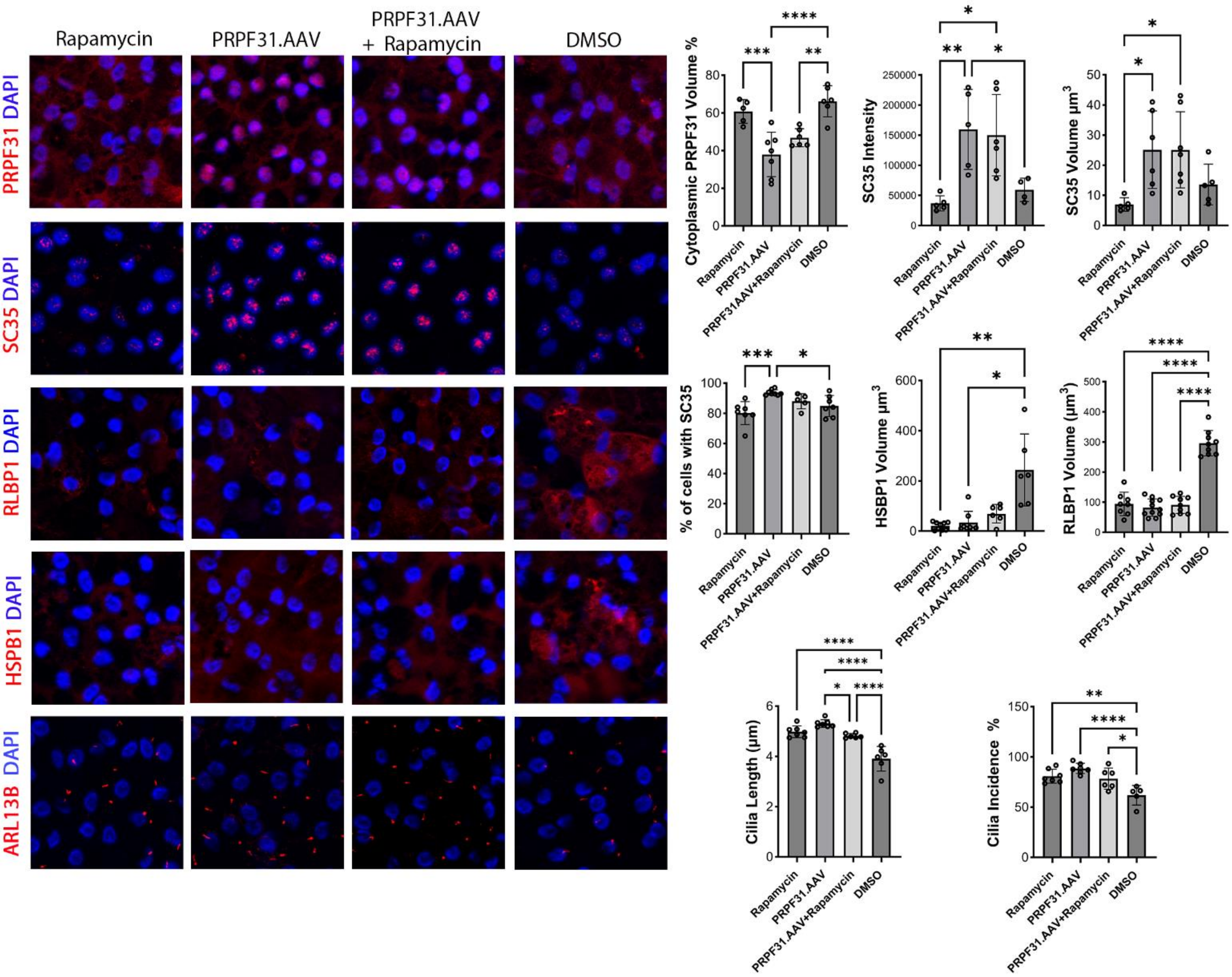

Figure S3: Comparative Evaluation of PRPF31 Augmentation and Rapamycin-Induced Autophagy in Mature RP11-RPE Cells.

Top Panel: Representative Immunofluorescence Imaging. Micrographs illustrate the therapeutic effects of *PRPF31*.AAV, Rapamycin, or combinatorial treatment on mature RP11-RPE. Markers include:

- Nuclear Homeostasis: PRPF31 (red) and SC35 (nuclear speckles; red).
- Proteotoxic Stress: RLBP1 and HSPB1 cytoplasmic aggregates (red).
- Ciliary Architecture: ARL13B (red).

Nuclei were counterstained with DAPI (blue). *PRPF31* augmentation successfully restored nuclear localisation, reorganised speckle morphology, cleared cytoplasmic aggregates, and lengthened primary cilia. In contrast, Rapamycin treatment alone only partially reduced the aggregate burden and provided no additive benefit when combined with *PRPF31*.AAV. Scale bars: 25 µm.

Right Panel: Quantitative Phenotypic Analysis. Comprehensive quantification of the rescue effects across all treatment groups. Metrics include PRPF31 volume (%), SC35 volume (µm3) and

intensity, percentage of SC35+ cells (%), RLBP1 and HSPB1 aggregate volume (μm3), and cilia length (μm) and incidence (%).

Statistical Analysis: Data are presented as mean ± SD (n = 10 representative images per marker per condition). Statistical significance was determined by one-way ANOVA followed by Tukey's multiple comparisons test. Source Data are provided in the Source Data file.

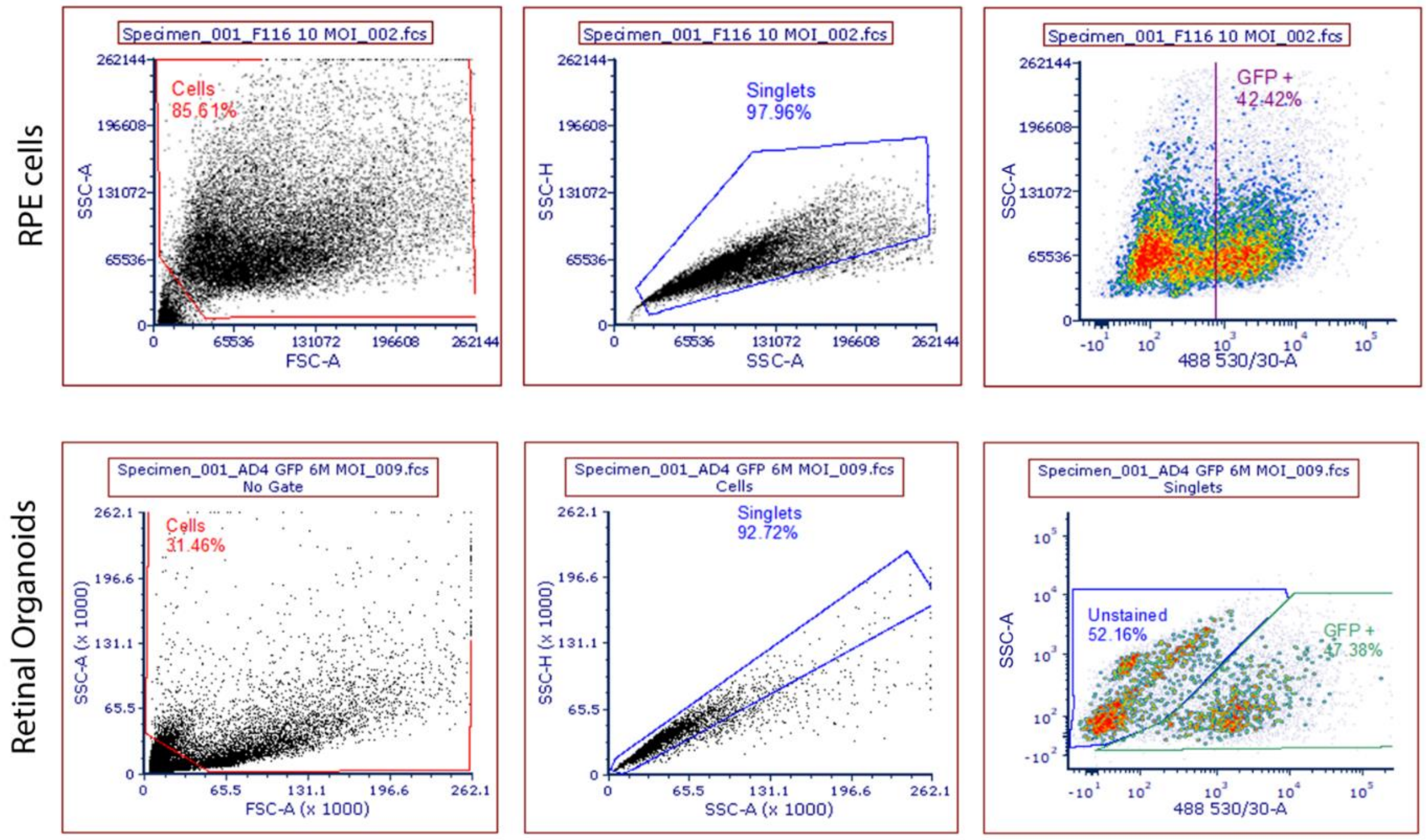

Figure S4: Representative flow cytometry gating strategies for RPE cells and retinal organoids. Gating strategy showing sequential identification of cells and single cells, followed by GFP-positive gating of cells in RPE cells (top panel) and retinal organoids (bottom panel). Percentages indicate the proportion of events within each gate.

Supplementary Table 1: Information of Patient and Wild-type controls used in this study.

Sample	Lab identifier	Age at biopsy	Age of onset of any symptoms	Sex	Type	Genotype	Phenotype
WT1	SB-Ad2	51	-	M	-	Wild type	Unaffected
WT3	SB-Ad4	68	-	M	-	Wild type	Unaffected
RP11S1	F116	69	10	F	PRPF31	c.1115_1125 del11	Severe - Presented with night blindness aged 10yrs old with decreased central vision by 30yrs. Cataract requiring surgery in 50s. Current visual acuity 6/60 and 6/24. Bilateral, extensive bone spicule pigmentation, attenuated arterioles and pale optic discs with small, preserved islands of RPE in macula.
RP11VS	F119	45	10	M	PRPF31	c.1115_1125 del11	Very severe - Presented with night blindness aged 10yrs old with decreased central vision by 30yrs. Also has right optic nerve hypoplasia. Current visual acuity - No perception of light right eye t and 6/18 left. Bilateral extensive bone spicule pigmentation, attenuated arterioles and pale optic discs and visual field restricted to around 5 degrees from fixation.
RP11S3	F150	50	15	F	PRPF31	c. 522_527+IVS+1 to +10	Severe – presented with night blindness aged 15yrs old with decreased central vision by the age of 30 years. Current visual acuities 6/18 and 6/38 with a refraction of -2 and bilateral posterior subcapsular cataracts.

Supplementary Table 2: Primer sequences used in this study.

Name	Forward	Reverse
Endogenous PRPF31	TCGTATATGGCGGGAAGTCC	CAGAACCCGATCCTAGCCC
Total PRPF31	GATTACATCCGCACGGTCAAG	CGACAGCTGCTGCCCCTG
AAV PRPF31	AAGGTCAAGGGCGAGAAGAG	GGGAGGTGTGGGAGGTTTT
AAV specific (Large T Antigen)	GGCCGCGACTCTAGATCATA	GGGAGGTGTGGGAGGTTTT
GAPDH	GTCTCCTCTGACTTCAACAGCG	ACCACCCTGTTGCTGTAGCCAA

Supplementary Table 3: Table of antibody details used in this study.

**Primary antibodies**				
**Name**	**Dilution**	**Clone**	**Company/ catalogue number**	**Lot number**
Anti-PRPF31	1 in 250	Rabbit	ProteinTech / 27750-1-AP	55616
Anti-SC-35	1 in 50	Mouse	Santa-cruz Biotechnology / sc-53518	D0720
Anti-Ezrin	1 in 100	Rabbit	Proteintech / 26056-1-AP	
Anti-Coll IV	1 in 400	Rabbit	Abcam / ab6586	GR3350938-1
Anti-Recoverin	1 in 1000	Rabbit	Millipore / AB5585	3099956
Anti-RLBP1 (CRALBP)	1 in 250	Mouse	Abcam / ab15051	GR3388978-2
Anti-HSPB1	1 in 250	Mouse	Aviva systems Biology / OASG03654	7485801
Anti-ARL13B	1 in 1000	Rabbit	ProteinTech / 17711-1-AP	110244
Anti-ZO-1	1 in 50	Mouse	Thermo Scientific / 33-9100	VD300168
Anti-PRPF31	1 in 2000	Rabbit	Bethyl Laboratories / A303-919A	1
Anti-GFP	1 in 2000	Mouse	Invitrogen / MA5-15256	XH352366
Anti-GAPDH	1 in 2000	Mouse	Santa Cruz Biotechnology / sc-47724	I2418
Anti-phospho-SF3B1	1 in 1000	Rabbit	Home-made	N/A
Anti-β-tubulin	1 in 2000	Mouse	Santa Cruz Biotechnology / sc-47778	L1520
**Secondary antibodies**				
**Name**	**Dilution**		**Company/Catalogue number**	
Donkey anti-Mouse IgG (H+L), Alexa Fluor™ 546	1 in 800		Invitrogen / A10036	1622582
Donkey anti-Rabbit IgG (H+L), Alexa Fluor™ 546	1 in 800		Invitrogen / A10040	2411581
Donkey anti-Rabbit IgG (H+L), Alexa Fluor™ Plus 647	1 in 800		Invitrogen / A32795	3231880
Goat Anti-Mouse IgG (H+L), Cy3	1 in 800		Jackson Immuno Research / 115-165-003	157282
Polyclonal Swine Anti-Rabbit Immunoglobulins/ HRP	1 in 2000		Agilent Dako/ P0399	41640456
Rabbit Anti-Mouse Immunoglobulins/ HRP	1 in 2000		Agilent Dako/ P0260	41326080

Supplementary Table 4: Table of software according to Cell Press Key Resources

Software and algorithms		
FastQC	Babraham Institute	www.bioinformatics.babraham.ac.uk/projects/fastqc
MultiQC	DOI: 10.1093/bioinformatics/btw354	https://multiqc.info/
STAR aligner	DOI: 10.1093/bioinformatics/bts635	https://github.com/alexdobin/STAR
featureCounts (Subread package)	DOI: 10.1093/bioinformatics/btt656	http://subread.sourceforge.net/
DESeq2	DOI: 10.1186/s13059-014-0550-8	https://bioconductor.org/packages/release/bioc/html/DESeq2.html
EnhancedVolcano	DOI: 10.18129/B9.bioc.EnhancedVolcano	https://bioconductor.org/packages/release/bioc/html/EnhancedVolcano.html
rMATs turbo	DOI: 10.1073/pnas.1419161111	https://github.com/Xinglab/rmats-turbo
clusterProfiler	DOI: 10.1016/j.xinn.2021.100141	https://bioconductor.org/packages/release/bioc/html/clusterProfiler.html
