# Supplementary material for: Cell-autonomous restoration of splicing homeostasis and RP11 phenotype in patient-derived RPE and retinal organoids by PRPF31.AAV gene therapy
Source: Nat Commun. 2026 Sep 30;17:10365. doi: 10.1038/s41467-026-78065-z (PMC13627695; doi:10.1038/s41467-026-78065-z)
Supplement: Supplementary file 2 [file 41467_2026_78065_MOESM2_ESM.pdf]

Description of Additional Supplementary Files

Supplementary Data 1: Differential gene expression in RPE samples and retinal organoids treated with PRPF31.AVV versus GFP.AAV.

Supplementary Data 2: Differential exon usage in retinal organoids and RPE cells treated with PRPF31.AAV versus GFP.AAV controls.

Supplementary Data 3: Differential protein abundance between PRPF31.AAV and GFP.AAV RPE cells and associated GO analysis for biological processes and cellular components.

Supplementary Data 4: Differential protein abundance between PRPF31.AAV and GFP.AAV retinal organoids and associated GO analysis for biological processes and cellular components.
