# Supplementary material for: Cell-autonomous restoration of splicing homeostasis and RP11 phenotype in patient-derived RPE and retinal organoids by PRPF31.AAV gene therapy
Source: Nat Commun. 2026 Sep 30;17:10365. doi: 10.1038/s41467-026-78065-z (PMC13627695; doi:10.1038/s41467-026-78065-z)
Supplement: Supplementary file 3 [file 41467_2026_78065_MOESM7_ESM.pdf]

nature portfolio

Corresponding author(s): Majlinda Lako

Last updated by author(s): 14/08/2026

Reporting Summary

Nature Portfolio wishes to improve the reproducibility of the work that we publish. This form provides structure for consistency and transparency in reporting. For further information on Nature Portfolio policies, see our Editorial Policies and the Editorial Policy Checklist.

Statistics

For all statistical analyses, confirm that the following items are present in the figure legend, table legend, main text, or Methods section.

n/a	Confirmed	
☐	☒	The exact sample size (*n*) for each experimental group/condition, given as a discrete number and unit of measurement
☐	☒	A statement on whether measurements were taken from distinct samples or whether the same sample was measured repeatedly
☐	☒	The statistical test(s) used AND whether they are one- or two-sided<br>*Only common tests should be described solely by name; describe more complex techniques in the Methods section.*
☒	☐	A description of all covariates tested
☐	☒	A description of any assumptions or corrections, such as tests of normality and adjustment for multiple comparisons
☐	☒	A full description of the statistical parameters including central tendency (e.g. means) or other basic estimates (e.g. regression coefficient) AND variation (e.g. standard deviation) or associated estimates of uncertainty (e.g. confidence intervals)
☐	☒	For null hypothesis testing, the test statistic (e.g. *F*, *t*, *r*) with confidence intervals, effect sizes, degrees of freedom and *P* value noted<br>*Give P values as exact values whenever suitable.*
☒	☐	For Bayesian analysis, information on the choice of priors and Markov chain Monte Carlo settings
☒	☐	For hierarchical and complex designs, identification of the appropriate level for tests and full reporting of outcomes
☒	☐	Estimates of effect sizes (e.g. Cohen's *d*, Pearson's *r*), indicating how they were calculated

Our web collection on statistics for biologists contains articles on many of the points above.

Software and code

Policy information about availability of computer code

Data collection	No software used
Data analysis	FastQC www.bioinformatics.babraham.ac.uk/projects/fastqc<br>MultiQC https://multiqc.info/<br>STAR aligner https://github.com/alexdobin/STAR<br>featureCounts (Subread package) http://subread.sourceforge.net/<br>DESeq2 https://bioconductor.org/packages/release/bioc/html/DESeq2.html<br>EnhancedVolcano https://bioconductor.org/packages/release/bioc/html/EnhancedVolcano.html<br>rMATs turbo https://github.com/Xinglab/rmats-turbo<br>DEXSeq https://bioconductor.org/packages/release/bioc/html/DEXSeq.html<br>clusterProfiler https://bioconductor.org/packages/release/bioc/html/clusterProfiler.html<br>Huygens Essential: https://svi.nl/Huygens-Essential<br>Fiji (ImageJ): https://imagej.net/software/fiji/<br>https://github.com/eltonjrv/bioinfo.scripts/blob/master/filter_rMATs.pl

For manuscripts utilizing custom algorithms or software that are central to the research but not yet described in published literature, software must be made available to editors and reviewers. We strongly encourage code deposition in a community repository (e.g. GitHub). See the Nature Portfolio guidelines for submitting code & software for further information.

Data

Policy information about availability of data

All manuscripts must include a data availability statement. This statement should provide the following information, where applicable:

- Accession codes, unique identifiers, or web links for publicly available datasets
- A description of any restrictions on data availability
- For clinical datasets or third party data, please ensure that the statement adheres to our policy

The RNA-Seq of all samples included in this study were uploaded GEO under the accession number GSE309908 (https://www.ncbi.nlm.nih.gov/geo/query/acc.cgi?acc=GSE309908). The proteomics data have been deposited to PRIDE under the accession number PXD076430 (https://proteomecentral.proteomexchange.org/ui?pxid=PXD076430).

Research involving human participants, their data, or biological material

Policy information about studies with human participants or human data. See also policy information about sex, gender (identity/presentation), and sexual orientation and race, ethnicity and racism.

Reporting on sex and gender	iPSCs used in this study were derived from three males and two females. There was no biological reason for investigating the role of sex on disease.
Reporting on race, ethnicity, or other socially relevant groupings	No such categorisation was used in our analysis.
Population characteristics	See above.
Recruitment	Participants were recruited based on clinical phenotype and the presence of mutation in the target gene (PRPF31).
Ethics oversight	Informed consent for research studies, according to the protocols approved by Yorkshire and the Humber Research Ethics Committee (REC ref: 03/362), was obtained for all study participants.

Note that full information on the approval of the study protocol must also be provided in the manuscript.

Field-specific reporting

Please select the one below that is the best fit for your research. If you are not sure, read the appropriate sections before making your selection.

☒ Life sciences ☐ Behavioural & social sciences ☐ Ecological, evolutionary & environmental sciences

For a reference copy of the document with all sections, see nature.com/documents/nr-reporting-summary-flat.pdf

Life sciences study design

All studies must disclose on these points even when the disclosure is negative.

Sample size	No sample size calculation was performed. To determine the optimal number of biological replicates for experiments in the current study, we performed statistical power calculations on data obtained from our previous PRPF31 study (PMID: 30315276). The power calculations indicated that three biological replicates per experimental group were sufficient for statistical power >0.8 associated with type 1 error rate α=0.05.
Data exclusions	No data was excluded. Some iPSC lines do not efficiently differentiate into certain tissues (e.g. RP11S3 into retinal organoids (RO), RP11S1 into retinal pigment epithelium (RPE)). This placed a constraint on the number of iPSC lines that could be used for experiments, particularly those requiring a large amount of biological material.
Replication	Biological triplicates were performed for all experiments, apart WB for retinal organoids where the large amount of AAVs required for each transduction made it impossible to perform more than 2 experiments, resulting in two biological replicates.
Randomization	Groups formed depending on genotype.
Blinding	Blinding is not relevant to this study. This is a descriptive study and the experiments and analysis were designed to minimise risk of subjective interpretation.

Reporting for specific materials, systems and methods

We require information from authors about some types of materials, experimental systems and methods used in many studies. Here, indicate whether each material, system or method listed is relevant to your study. If you are not sure if a list item applies to your research, read the appropriate section before selecting a response.

Materials & experimental systems

n/a	Involved in the study
☐	☒ Antibodies
☐	☒ Eukaryotic cell lines
☒	☐ Palaeontology and archaeology
☒	☐ Animals and other organisms
☒	☐ Clinical data
☒	☐ Dual use research of concern
☒	☐ Plants

Methods

n/a	Involved in the study
☒	☐ ChIP-seq
☐	☒ Flow cytometry
☒	☐ MRI-based neuroimaging

Antibodies

Antibodies used

Antibody - Species - Supplier - Catalog number/ Lot number - Dilution
Anti-PRPF31 - Rabbit - ProteinTech - 27750-1-AP / 00055616 - 1 in 250
Anti-SC-35 - Mouse - Santa-cruz Biotechnology - sc-53518 / D0720 - 1 in 50
Anti-Ezrin - Rabbit - Proteintech - 26056-1-AP 1 in 100
Anti-Coll IV - Rabbit - Abcam - ab6586 / GR3350938-1 - 1 in 400
Anti-Recoverin - Rabbit - Millipore - AB5585 / 3099956 - 1 in 1000
Anti-RLBP1 (CRALBP) - Mouse - Abcam - ab15051 / GR3388978-2 - 1 in 250
Anti-HSPB1 - Mouse - Aviva systems Biology - OASG03654 / 7485801 - 1 in 250
Anti-ARL13B - Rabbit - ProteinTech - 17711-1-AP / 00110244 - 1 in 1000
Anti-ZO-1 - Mouse - Thermo Scientific - 33-9100 / VD300168 - 1 in 50
Anti-PRPF31 - Rabbit - Bethyl Laboratories - A303-919A / 1 - 1 in 2000
Anti-GFP - Mouse - Invitrogen - MA5-15256 / XH352366 - 1 in 2000
Anti-GAPDH - Mouse - Santa Cruz Biotechnology - sc-47724 / I2418 - 1 in 2000
Anti-phospho-SF3B1 - Rabbit - Home-made N/A - 1 in 1000
Anti- β-tubulin - Mouse - Santa Cruz Biotechnology - sc-47778 / L1520 - 1 in 2000

Donkey anti-Mouse IgG (H+L), Alexa Fluor™ 546 - Mouse - Invitrogen - A10036 / 1622582 - 1 in 800
Donkey anti-Rabbit IgG (H+L), Alexa Fluor™ 546 - Rabbit - Invitrogen - A10040 / 2411581 - 1 in 800
Donkey anti-Rabbit IgG (H+L), Alexa Fluor™ Plus 647 - Rabbit - Invitrogen - A32795 / 3231880 - 1 in 800
Goat Anti-Mouse IgG (H+L), Cy3 - Mouse - Jackson Immuno Research - 115-165-003 / 157282 - 1 in 800
Polyclonal Swine Anti-Rabbit Immunoglobulins/HRP - Rabbit - Agilent Dako - P0399 / 41640456 - 1 in 2000
Rabbit Anti-Mouse Immunoglobulins/HRP - Mouse - Agilent Dako - P0260 / 41326080 - 1 in 2000

Validation

Antibody Validation and cited examples of their use from manufacturer's website and published studies:
PRPF31 antibody (Proteintech) used for immunofluorescence experiments: PMID: 41864154, PMID: 42105440.
SC-35 antibody used for immunofluorescence experiments, cited in 10 publications, PMID: 20519584.
ARL13B used in immunofluorescence experiments: Antibody validated in PMID: 36764291, PMID: 28562594 (879 publications in total citing this antibody in immunofluorescence experiments).
Ezrin antibody used for immunofluorescence experiment validated in PMID: 35305541 and PMID: 28938000 (24 publications in manufacturer's website). Antibody validation provided by the manufacturer website: https://www.ptglab.com/products/Ezrin-Antibody26056-1-AP.htm.
CRALBP used for immunofluorescence experiments: antibody validated in PMID:28922560, PMID: 27506453, PMID: 22892561.
Collagen IV used for immunofluorescence experiments, cited in 1,127 publications (PMID: 28158775), validated by supplier.
Recoverin antibody used for immunofluorescence experiments. Validated by supplier, 17 publications with applications in IF and research area of Stem Cells and Developmental Biology (PMID: 35330501).
HSPB1 antibody used for immunofluorescence experiments. Antibody validated by supplier, cited in PMID: 35297555.
ZO-1 used for immunofluorescence experiments. Antibody validated by supplier (430 publications in total citing this antibody in IF experiments) and in PMID: 29487356, PMID: 29445080.
PRPF31 (Bethyl Laboratories) used for Western blot experiments. Validated by supplier and cited in 2 publications (PMID: 34789764 and PMID: 31010829).
GFP antibody used for immunofluorescence experiments. Validated in PMID: 35228412, PMID: 31964834 and PMID: 33514733; cited in 33 publications with applications in IF.
GAPDH antibody used for western blot experiments. Antibody validated by supplier, cited in 3,396 publications.
SF3B1 antibody used for immunofluorescence experiments, validated by Dr Sina Mozaffari Jovin.
β-tubulin antibody used for Western blot experiments. Validated by supplier, cited in 656 publications for use in western blot (PMID: 33986153, PMID:31300274).

Plants

Seed stocks

Report on the source of all seed stocks or other plant material used. If applicable, state the seed stock centre and catalogue number. If plant specimens were collected from the field, describe the collection location, date and sampling procedures.

Novel plant genotypes

Describe the methods by which all novel plant genotypes were produced. This includes those generated by transgenic approaches, gene editing, chemical/radiation-based mutagenesis and hybridization. For transgenic lines, describe the transformation method, the number of independent lines analyzed and the generation upon which experiments were performed. For gene-edited lines, describe the editor used, the endogenous sequence targeted for editing, the targeting guide RNA sequence (if applicable) and how the editor was applied.

Authentication

Describe any authentication procedures for each seed stock used or novel genotype generated. Describe any experiments used to assess the effect of a mutation and, where applicable, how potential secondary effects (e.g. second site T-DNA insertions, mosiacism, off-target gene editing) were examined.

Flow Cytometry

Plots

Confirm that:

☒ The axis labels state the marker and fluorochrome used (e.g. CD4-FITC).

☒ The axis scales are clearly visible. Include numbers along axes only for bottom left plot of group (a 'group' is an analysis of identical markers).

☒ All plots are contour plots with outliers or pseudocolor plots.

☒ A numerical value for number of cells or percentage (with statistics) is provided.

Methodology

Sample preparation

All information on sample preparation is presented in the methods section.

RPE cells were washed with PBS and were detached from the wells using 200µl of TrypIE Select (10X) for 15 minutes. Then, RP11-RPE cells were resuspended in flow buffer (PBS with 2% FBS) and were transferred into Eppendorf tubes followed by centrifugation at 300 × g. RPE cells were washed 3 times with PBS, and cell pellets were resuspended in flow buffer.

Retinal organoids day 150 were collected and dissociated using the Neurosphere Dissociation Kit (Miltenyi Biotec). Cells were resuspended in 1% Bovine Serum Albumin (in PBS) and incubated with CD73-APC- conjugated surface marker for 30 minutes at 4oC. Then, samples were washed with 1x Binding Buffer (eBioscience) and resuspened in Flow buffer (PBS with 2% FBS). Cell pellets were washed 3 times with PBS and resuspended in flow buffer.

Instrument

Samples were analysed on a BD LSRFortessa X-20 cell analyser.

Software

RPE and RO samples were analysed using FCS Express 7.

Cell population abundance

At least 10,000 cells were analyzed from each sample.

Gating strategy

Initial gating of debris was performed using FSC-A vs SSC-A. In analysis, singlets were identified using SSC-A vs SSC-H. Then fluorescence was measured using the excitation and filter indicated.

☒ Tick this box to confirm that a figure exemplifying the gating strategy is provided in the Supplementary Information.
