# Supplementary material for: Cell-autonomous restoration of splicing homeostasis and RP11 phenotype in patient-derived RPE and retinal organoids by PRPF31.AAV gene therapy
Source: Nat Commun. 2026 Sep 30;17:10365. doi: 10.1038/s41467-026-78065-z (PMC13627695; doi:10.1038/s41467-026-78065-z)
Supplement: Supplementary file 4 [file 41467_2026_78065_MOESM8_ESM.pdf]

nature portfolio

Peer Review File

Cell-autonomous restoration of splicing homeostasis and RP11 phenotype in patient-derived RPE and retinal organoids by PRPF31.AAV gene therapy

Corresponding Author: Professor Majlinda Lako

This file contains all reviewer reports in order by version, followed by all author rebuttals in order by version.

Version 0:

Reviewer comments:

Reviewer #1

(Remarks to the Author)
In the study titled “Cell-autonomous restoration of splicing homeostasis and RP11 phenotype in patient-derived RPE and retinal organoids by PRPF31.AAV gene therapy” the authors demonstrate use of patient iPSC-derived RPE and retinal organoids to evaluate pathobiology of PRPF31 associated retinal degeneration and to evaluate efficacy of a novel gene-based therapeutic. They demonstrate that AAV41 mediated PRPF31 augmentation restores nuclear localization, SC35+ nuclear speckle reorganization, and enhancement of p-SF3B1⁺ active spliceosome foci. In addition, they go on to demonstrate global rescue of splicing activity, upregulation of phagocytosis, increased protein aggregate clearance pathways, and mitochondrial proteins. These molecular shifts facilitated the clearance of proteotoxic cytoplasmic aggregates and reversed key functional deficits; specifically, they reinforced RPE apical-basal polarity, restored phagocytic capacity and normal ciliary morphology and incidence, and boosted light-evoked activity in photoreceptors. Importantly the authors demonstrate that phenotypic rescue was achievable in mature RPE, supporting a broad clinical window for intervention.

In general, the manuscript is well written, presents a very large body of data, and will be of interest to a broad readership.

To strengthen the manuscript the reviewer has the following recommendations.
1. Supplemental figure 1 there appears to be diffuse/poor expression of zo1 resulting from poor differentiation and or partial EMT. As the developmental state of the RPE culture may alter transduction efficiency it is important that mature cultures of pigmented RPE with the proper hexagonal morphology be transduced. Similarly, panel F does not appear to agree with panel E. Specifically, in panel E there would appear to be far less than 65-70% GFP expression. This could result from gating issues with FACs or representative image selection. The reviewer recommends showing lower mag wide field of view images of RPE that support the data in F.
2. Figure 1C, inclusion of a wildtype, or isogenic control line demonstrating normal expression pattern would be useful. In addition, the authors should comment on what appears to be increased SC35 expression/speckle following AAV-GFP transduction (Figure 1C), and the fact the at DAPI is diffuse and does not appear to be restricted to the nuclei (Figure 1F).
3. Figure 2C there appears to be significant PRPF staining outside of DAPI positive cells. Inclusion of structural stains demonstrating that PRPF is intracellular and which cell type it is associated with would be useful. Likewise, a normal or isogenic control demonstrating normal expression levels and patterns would be useful. Finally Figure 2D the morphology of the organoid appears to be abnormal. Replacing with a well laminated organoid stained with photoreceptor cell markers is recommended.
4. Figure 3 and 7A&B, as indicated above cells do not appear to be hexagonal. Similarly, RLBP expression is not overly compelling. Low mag images demonstrating correct RPE cell morphology, pigmentation, etc., and western blot analysis demonstrating reduced expression of RLBP and HSP would be helpful.
5. Data presented in Figure S3 is compelling. The author might consider including this as a primary figure.
6. Figure 4A and 7D, western blot analysis demonstrating internalization of POS using membrane vs cytoplasmic fractions would be helpful. As presented it is difficult for the reader to determine if POS are phagocytosed or simply stuck to the surface of the cells.
7. Figure 4B-C, the data are convincing however, it is difficult for the reviewer to understand exactly how the study was done. Addition of details to the figure legend pertaining to experimental design would be useful (e.g., a schematic demonstrating how the experiment was performed would be helpful).

Minor edits
1. “double dose (Figure S1D).” the () are bold.
2. Figure 3 the label for panel C is overlapping the stain label and difficult to see.
3. 344 (Figure 6A, B). the) is bold.

Reviewer #2

(Remarks to the Author)
The authors used RP11 patient ips cells derived RPE cells and retinal organoids to elucidate the functional mechanisms of AAV-PRPF31's beneficial effects. The authors demonstrated that AAV-PRPF31 treatment restored nuclear speckle size, decreased accumulation of cytotoxic aggregates, rescued ciliary defects, improved phagocytic activities, rescued transcriptomic defects, and reshaped proteomic landscape in patient isp-derived RPE cells and retinal organoid. The data demonstrated the efficiency of AAV-PRPF31 in rescuing PRPF31 mutant mediated deficits and the underling mechanisms. As the the PRPF31 mutants caused deficits in ips-derived RPE cells and retinal organoid have been previous reported by the group, the rescuing effects of AAV-PRPF31 have previous partially reported by the groups, the authors also did not examine the rescuing effects of AAV-PRPF31 in PRPF31 conditional knockout or knockin mouse models. The other group has reported AAV-mediated PRPF31 gene augmentation restored the retinal structure and function in a rapidly degenerating mouse model. Therefore, the manuscript lacks of enough novelty. I have some minor comments:
1. As PRPF31 mutants cause photoreceptor degeneration, the authors examined retinal ganglion cell response, is really related to PRPF function?
2. The authors described transcriptional changes in cholesterol and lipid metabolism, it may need to measure whether thee is any changes in levels of cholesterol and other lipids.
3. In Method section, the authors described two WT ips cell lines and three patients ips cell lines, but the authors only presented data about untreated, AAV-GFP-treated and AAV-PRPF31-treated cells, there was no information about WT-derived RPE/retinal organoid, or any information which patients ips-derived RPE/retinal organoid.

Reviewer #3

(Remarks to the Author)
The paper by Elia et al investigates, by using patient-specific iPSC-derived RPE cells and retinal organoids (RO), through which mechanisms the AAV-mediated administration of the PRPF31 gene exerts a therapeutic effect in the autosomal dominant form of RP termed RP11. The Authors previously observed in iPSC-derived RPE and RO from RP11 patients global splicing impairment, spliceosomal complexes alterations with PRPF31 protein mislocalization to the cytoplasm and aggregate formation and eventually defective phagocytosis and abnormal ciliogenesis. Previous studies also reported on a beneficial effect of AAV-mediated administration in either iPSC-derived RPE or RO from patients but without investigating the effects on the key pathogenic events involved in PRPF31 dysfunction.
In this report, the Authors show that the use of a single AAV serotype (ShH10(Y445F) is effective in counteracting most of the functional abnormalities caused by PRPF31 mutation both in RPE and photoreceptor cells, as also assessed by OMICS approaches. Moreover, they also observed some beneficial effects also in mature RP11-RPE cells, which is of high relevance for future translational goals.
This is a solid paper, with high translational value for the future set up of a gene therapy treatment of RP11. It is well written and the data are overall clearly presented. I only have the following (mostly minor) remarks not necessarily sorted by importance:

1) The Authors should more clearly indicate also in the Results (and not only in the Methods) the identity of the promoter used for their AAV constructs.
2) Figures 1-2: Bottom left panel in Figure 1A seems to be repeated in Figure 2A, again as bottom left panel but in both cases without any comment in the text. The Authors should consider leaving this panel only in one place, preferentially in Figure 2 when they also compare PRPF31 protein expression values between iPSC-RPE and RO (see lines 194-195). In any case, some further comment on the results shown in these panels is needed.
3) Lines 177-179, statement “however, a residual cytoplasmic PRPF31 fraction persisted in AAV-transduced cells (23.5%) compared with a negligible cytoplasmic signal in wild-type controls (11). Are the Authors referring exclusively to a previous publication here? The way it is presented seem to indicate results shown in the paper.
4) Transcriptomic and proteomic analyses: the Authors should provide more details on the number of samples analyzed both for the RNAseq and proteomic analyses. Also, did they use in both cases RP11-RPE and RP11-RO from all the three patients available (mentioned in the Methods)?
5) Transcriptomic analysis: The Authors describe a number of differential splicing events between PRPF31.AAV-treated and GFP.AAV-treated samples, both RP11-RPE (241 events) and RP11-RO (261 events) (page 10). It is not clear whether there are alternative splice events that are specific (enriched) in control(GFP)-treated cells vs. PRPF31-treated ones. If so, which are the numbers? A more detailed explanation of these analyses is needed.
6) Transcriptomic analysis: again, when reporting the results of the enrichment analysis of the genes affected by changes in splicing pattern, the Authors do not mention whether some of these genes also display differential gene expression (Figure 5A-B).

Version 1:

Reviewer comments:

Reviewer #1

(Remarks to the Author)
The authors have addressed all of this reviewers previous comments. They present an outstanding study with conclusions supported by a large body of compelling high quality data. This reviewer has no further suggestions!

Reviewer #2

(Remarks to the Author)
The authors have addressed my comments. I have no further concerns.

Reviewer #3

(Remarks to the Author)
The Authors properly addressed all the issues raised

Open Access This Peer Review File is licensed under a Creative Commons Attribution 4.0 International License, which permits use, sharing, adaptation, distribution and reproduction in any medium or format, as long as you give appropriate credit to the original author(s) and the source, provide a link to the Creative Commons license, and indicate if changes were made.
In cases where reviewers are anonymous, credit should be given to 'Anonymous Referee' and the source.
The images or other third party material in this Peer Review File are included in the article's Creative Commons license, unless indicated otherwise in a credit line to the material. If material is not included in the article's Creative Commons license and your intended use is not permitted by statutory regulation or exceeds the permitted use, you will need to obtain permission directly from the copyright holder.
To view a copy of this license, visit https://creativecommons.org/licenses/by/4.0/

Reviewer #1

In the study titled "Cell-autonomous restoration of splicing homeostasis and RP11 phenotype in patient-derived RPE and retinal organoids by PRPF31.AAV gene therapy" the authors demonstrate use of patient iPSC-derived RPE and retinal organoids to evaluate pathobiology of PRPF31 associated retinal degeneration and to evaluate efficacy of a novel gene-based therapeutic. They demonstrate that AAV41 mediated PRPF31 augmentation restores nuclear localization, SC35+ nuclear speckle reorganization, and enhancement of p-SF3B1+ active spliceosome foci. In addition, they go on to demonstrate global rescue of splicing activity, upregulation of phagocytosis, increased protein aggregate clearance pathways, and mitochondrial proteins. These molecular shifts facilitated the clearance of proteotoxic cytoplasmic aggregates and reversed key functional deficits; specifically, they reinforced RPE apical-basal polarity, restored phagocytic capacity and normal ciliary morphology and incidence, and boosted light-evoked activity in photoreceptors. Importantly the authors demonstrate that phenotypic rescue was achievable in mature RPE, supporting a broad clinical window for intervention.

In general, the manuscript is well written, presents a very large body of data, and will be of interest to a broad readership.

To strengthen the manuscript the reviewer has the following recommendations.

1. Supplemental figure 1 there appears to be diffuse/poor expression of zo1 resulting from poor differentiation and or partial EMT. As the developmental state of the RPE culture may alter transduction efficiency, it is important that mature cultures of pigmented RPE with the proper hexagonal morphology be transduced.

We thank the reviewer for this important comment. We have improved the quality of Figure S1E. We would also like to highlight that tight junctions, as indicated by ZO-1 staining, are disrupted in RP11-RPE cells compared with control cells. This observation is consistent with previously reported alterations in tight and adherens junction-associated proteins in the retina of animal models of autosomal dominant retinitis pigmentosa. This finding has been reported in our previous study (Georgiou et al., Clinical and Translational Medicine, 2022, 12: e759).

Similarly, panel F does not appear to agree with panel E. Specifically, in panel E there would appear to be far less than 65-70% GFP expression. This could result from gating issues with FACs or representative image selection. The reviewer recommends showing lower mag wide field of view images of RPE that support the data in F.

We have reanalysed the data corresponding to Figures S1E and S1F and confirm that the quantification presented in Figure S1F is accurate. Additionally, we have included a separate image in Figure S1E to more clearly illustrate the GFP-positive cells

2. Figure 1C, inclusion of a wildtype, or isogenic control line demonstrating normal expression pattern would be useful.

This control has been added (See revised Figure 1C). Importantly, a couple of sentences to reflect these findings have been added on page 6 as follows: The restored localisation pattern closely mirrored that observed in wild-type RPE cells; however, a residual cytoplasmic PRPF31 fraction persisted in PRPF31.AAV-transduced cells (23.5%) compared with a negligible cytoplasmic signal in wild-type controls (4.4.%) (**Figure 1C**). A second note of "notably, SC35 immunostaining results appeared comparable to wild-type controls in PRPF31.AAV-treated RPE cells" has been added to the revised results, page 7.

In addition, the authors should comment on what appears to be increased SC35 expression/speckle following AAV-GFP transduction (Figure 1C).

We thank the reviewer for this important comment. We note that both AAV vectors increase the proportion of SC35-positive RPE cells, indicating that viral transduction is associated with a broader change in nuclear speckle positivity. However, only PRPF31.AAV increased SC35 staining intensity and volume, suggesting that PRPF31 supplementation specifically enhances splicing-factor enrichment or nuclear speckle maturation beyond the effect of transduction alone (revised results, page 6).

DAPI is diffuse and does not appear to be restricted to the nuclei (Figure 1F). We are a bit confused with this remark as Figure 1 does not contain an F panel.

3. Figure 2C there appears to be significant PRPF staining outside of DAPI positive cells. Inclusion of structural stains demonstrating that PRPF is intracellular and which cell type it is associated with would be useful.

We appreciate this comment and have used Recoverin as a pan-photoreceptor marker to assess PRPF31 staining specifically in photoreceptors. The apparent PRPF staining outside DAPI-positive regions is consistent with the specialised subcellular localisation of spliceosome components in photoreceptor cells. As documented in the literature on photoreceptor transcription and splicing machinery, spliceosomes in mammalian photoreceptors exhibit unique localisation patterns distinct from canonical nuclear staining [Cell Tissue Res 2015 Nov;362(2):281-94]. Our previous work directly supports this interpretation. We have shown that phosphorylated SF3B1 (p-SF3B1), a marker of active spliceosomes, and phosphorylated PRPF31 are increased in RP13 photoreceptors, with p-SF3B1 immunostaining revealing that active spliceosomes preferentially localise at the periphery of nuclei rather than in the central DAPI-dense chromatin regions [Atkinson et al. Nat Commun. 2024 Apr 11; 15:3138, Fig. 5C]. These p-SF3B1-marked spliceosomes colocalise with SC35-positive nuclear speckles, significantly accumulating in RP13 photoreceptors, consistent with defects in splicing kinetics [Atkinson et al. Nat Commun. 2024 Apr 11; 15:3138, Fig. 5C inset, 5D–E]. We have also demonstrated that RNA polymerase II (RNAPII), a marker for active transcription sites, localises within and around nuclear speckle clusters and at the periphery of nuclear chromatin in photoreceptors [Atkinson et al. Nat Commun. 2024 Apr 11; 15:3138, Fig. 5H]. Together, these findings demonstrate the presence of a unique splicing compartment at the nuclear periphery of human photoreceptor cells consisting of Cajal bodies, nuclear speckles, active transcription sites, and spliceosomes [PMC11009313, Fig. 5I], which justifies the appearance of PRPF31 staining outside the nuclei. We have added the following note in the revised results (page 7) "Notably, in photoreceptor cells, PRPF31 and other splicing components exhibit a distinct perinuclear localisation, with active spliceosomes preferentially accumulating at the periphery of nuclei rather than in the central DAPI-dense chromatin regions, colocalising with SC35-positive nuclear speckles (Atkinson et al. Nat Commun. 2024 Apr 11; 15:3138,). This perinuclear spliceosome compartment, which includes Cajal bodies, nuclear speckles, and active transcription sites, is a unique feature of photoreceptor splicing machinery that may account for PRPF staining patterns appearing outside central DAPI-positive regions (Derlig et al. Cell Tissue Res. 2015 Nov;362:281-94).

Likewise, a normal or isogenic control demonstrating normal expression levels and patterns would be useful.

This has been added; please refer to the revised Figure 2C. A note to reflect the similarity between PRPF31.AAV-treated ROs, and wild-type controls has been added in page 7 as follows: "This restoration of PRPF31 expression translated into structural improvements within the nucleus. We observed a significant increase in PRPF31 volume and intensity of SC35-stained nuclear speckles in PRPF31.AAV-treated ROs, suggesting a broad rescue of nuclear speckle integrity (Figure 2C), with values comparable to those observed in wild-type controls."

Finally Figure 2D the morphology of the organoid appears to be abnormal. Replacing with a well laminated organoid stained with photoreceptor cell markers is recommended. We thank the reviewer for this comment. New images of laminated ROs subjected to p-SF3B1 immunostaining are shown in the revised Figure 2D.

4. Figure 3 and 7A&B, as indicated above cells do not appear to be hexagonal. Similarly, RLBP expression is not overly compelling. Low mag images demonstrating correct RPE cell morphology, pigmentation, etc., and western blot analysis demonstrating reduced expression of RLBP and HSP would be helpful.

Bright-field images of PRPF31-RPE cells have been added to the revised Figure 3 and Figure 7 (now renumbered as Figure 8 following the inclusion of an additional figure as recommended by the reviewer). Regarding cell morphology, we refer the reviewer to our response to point 1 (ZO-1 immunostaining), which highlights the disruption of tight and adherens junctions in PRPF31-RPE cells. Reduced expression of RLPB and HSPB1 by western blot is now shown in Figure 3B.

5. Data presented in Figure S3 is compelling. The author might consider including this as a primary figure.
We thank the reviewer for this comment. Figure S3 has now become Figure 4.

6. Figure 4A and 7D, western blot analysis demonstrating internalization of POS using membrane vs cytoplasmic fractions would be helpful. As presented it is difficult for the reader to determine if POS are phagocytosed or simply stuck to the surface of the cells. We thank the reviewer for this helpful suggestion. We agree that additional details in the figure legend would improve clarity regarding the experimental design. We have added a schematic diagram illustrating the phagocytosis assay workflow (Figure 5A) and include

the following key details: The phagocytosis assay utilises pH-sensitive fluorescent particles (e.g., pHrodo-labeled substrates) that are non-fluorescent at neutral pH but fluoresce brightly only in acidic environments. Fluorescence is detected specifically once particles are internalised into phagocytic vesicles, as the intracellular phagosome undergoes rapid acidification following particle uptake. This pH-dependent fluorescence mechanism allows clear distinction between true phagocytosis (internalised particles) and particle adherence to the cell surface, as non-internalised particles remain in the neutral extracellular environment and do not generate a fluorescent signal. The assay thereby provides robust, background-reduced measurement of phagocytic engulfment detectable by fluorescence microscopy or flow cytometry. This revised text has also been added to the revised results, pages 8- 9.

Minor edits

1. “double dose (Figure S1D).” the () are bold. Corrected
2. Figure 3 the label for panel C overlaps the stain label and is difficult to see. Corrected
3. 344 (Figure 6A, B). the) is bold. Corrected

Reviewer #2

The authors used RP11 patient ips cells derived RPE cells and retinal organoids to elucidate the functional mechanisms of AAV-PRPF31's beneficial effects. The authors demonstrated that AAV-PRPF31 treatment restored nuclear speckle size, decreased accumulation of cytotoxic aggregates, rescued ciliary defects, improved phagocytic activities, rescued transcriptomic defects, and reshaped proteomic landscape in patient isp-derived RPE cells and retinal organoid. The data demonstrated the efficiency of AAV-PRPF31 in rescuing PRPF31 mutant mediated deficits and the underling mechanisms. As the the PRPF31 mutants caused deficits in ips-derived RPE cells and retinal organoid have been previous reported by the group, the rescuing effects of AAV-PRPF31 have previous partially reported by the groups, the authors also did not examine the rescuing effects of AAV-PRPF31 in PRPF31 conditional knockout or knockin mouse models. The other group has reported AAV-mediated PRPF31 gene augmentation restored the retinal structure and function in a rapidly degenerating mouse model. Therefore, the manuscript lacks of enough novelty. I have some minor comments:

1. As PRPF31 mutants cause photoreceptor degeneration, the authors examined retinal ganglion cell response, is really related to PRPF function?

We thank the reviewer for raising this important question. The examination of retinal ganglion cell (RGC) responses is directly relevant to assessing PRPF31 function and photoreceptor rescue, and we provide the following justification:

1. **RGCs are the functional readout of photoreceptor activity:** Photoreceptors (rods and cones) do not fire action potentials in response to light; instead, they generate graded hyperpolarisations. The electrical signals (spike activity) that convey visual information to the brain are generated exclusively by RGCs, which bear the sole responsibility of propagating visual stimuli to the brain through their axons that form the optic nerve. Therefore, RGC responses serve as the definitive functional readout of photoreceptor health and synaptic transmission.
2. **Signal transmission requires intact retinal circuitry:** Visual signals are relayed across two or more chemical synapses from photoreceptors through bipolar cells to RGCs. When photoreceptors degenerate due to *PRPF31* mutations, the entire retinal circuitry is disrupted, resulting in diminished RGC responses. Our previous work demonstrated that patient PRP31 retinal organoids exhibit disrupted functional signalling characterised by significantly diminished RGC responses, confirming that photoreceptor dysfunction directly impacts downstream RGC activity (Buskin et al. Nat Commun 2018 Oct 12;9:4234).
3. **RGC responses assess synaptic transmission and network integrity:** By measuring light-evoked RGC spike activity, we directly assess whether PRPF31 augmentation rescues not only individual photoreceptor function but also the overall functional integrity of the retinal circuitry. The increased number of spikes and the stronger increased activity change in PRPF31.AAV-transduced ROs demonstrate improved synaptic transmission between rescued photoreceptors and downstream RGCs.
4. **Standard approach in retinal degeneration research:** Functional assessment of RGC responses is a well-established method for evaluating photoreceptor rescue in retinal degenerative diseases. When photoreceptors die from degenerative diseases, surviving retinal networks are left without their major source of input, and RGC functional stability reflects the extent of photoreceptor preservation.

In summary: We examined RGC responses because they are the only retinal cells that generate action potentials in response to light stimulation, providing a quantitative measure of whether PRPF31 augmentation successfully rescues photoreceptor function

and restores synaptic transmission within the retinal network. The enhanced RGC responses we observed directly demonstrate that PRPF31 supplementation improves both photoreceptor function and downstream retinal circuitry integrity.

To clarify this in the text, we have added the following note: “We examined RGC responses because they are the only retinal cells that generate action potentials in response to light stimulation, providing a quantitative measure of whether PRPF31 augmentation successfully rescues photoreceptor function and restores synaptic transmission within the retinal network” (page 9 of the results section).

2. The authors described transcriptional changes in cholesterol and lipid metabolism, it may need to measure whether thee is any changes in levels of cholesterol and other lipids. We thank the reviewer for this valuable suggestion. Our RNA-seq analysis identified differential expression of genes involved in cholesterol metabolism and lipoprotein receptor-mediated endocytosis, including HMGCR, LDLR, LRP2, and CYP51A1. However, we did not directly measure cellular cholesterol or lipid levels in the present study. We agree that assessing cholesterol and lipid content would provide important functional validation of the observed transcriptional changes. We have added this point to the Discussion as a limitation of the current work and as an important direction for future studies aimed at determining whether the transcriptional alterations translate into measurable changes in lipid homeostasis. Note added on discussion “Although transcriptomic analysis revealed regulation of genes involved in cholesterol metabolism and lipid transport, we did not directly assess cellular cholesterol or lipid levels. Future studies incorporating lipidomic analyses and cholesterol quantification will be important to determine whether these transcriptional changes result in functional alterations in lipid homeostasis and contribute to the observed reduction in protein aggregation”, page 17.

3. In Method section, the authors described two WT ips cell lines and three patients ips cell lines, but the authors only presented data about untreated, AAV-GFP-treated and AAV-PRPF31-treated cells, there was no information about WT-derived RPE/retinal organoid, or any information which patients ips-derived RPE/retinal organoid.

We have used RP11S1, RP11S3, RP11VS, WT1 and WT3 for experiments in RPE cells and RP11S1, WT3 and WT1 for experiments in ROs. This information has been added to the methods section, page 20.

Reviewer #3

The paper by Elia et al investigates, by using patient-specific iPSC-derived RPE cells and retinal organoids (RO), through which mechanisms the AAV-mediated administration of the PRPF31 gene exerts a therapeutic effect in the autosomal dominant form of RP termed RP11. The Authors previously observed in iPSC-derived RPE and RO from RP11 patients global splicing impairment, spliceosomal complexes alterations with PRPF31 protein mislocalization to the cytoplasm and aggregate formation and eventually defective phagocytosis and abnormal ciliogenesis. Previous studies also reported on a beneficial effect of AAV-mediated administration in either iPSC-derived RPE or RO from patients but without investigating the effects on the key pathogenic events involved in PRPF31 dysfunction.

In this report, the Authors show that the use of a single AAV serotype (ShH10(Y445F) is effective in counteracting most of the functional abnormalities caused by PRPF31 mutation both in RPE and photoreceptor cells, as also assessed by OMICS approaches. Moreover, they also observed some beneficial effects also in mature RP11-RPE cells, which is of high relevance for future translational goals.

This is a solid paper, with high translational value for the future set up of a gene therapy treatment of RP11. It is well written and the data are overall clearly presented. I only have the following (mostly minor) remarks not necessarily sorted by importance:

1) The Authors should more clearly indicate also in the Results (and not only in the Methods) the identity of the promoter used for their AAV constructs.

We thank the reviewer for this comment. The information has been added in results page 5 and 6.

2) Figures 1-2: Bottom left panel in Figure 1A seems to be repeated in Figure 2A, again as bottom left panel but in both cases without any comment in the text. The Authors should consider leaving this panel only in one place, preferentially in Figure 2 when they also compare PRPF31 protein expression values between iPSC-RPE and RO (see lines 194-195). In any case, some further comment on the results shown in these panels is needed.

We thank the reviewer for this observation. The Western blot panel was included in both figures to facilitate interpretation of the PRPF31 protein expression data in each experimental context. However, we agree that this duplication is unnecessary.

Accordingly, we have removed the duplicated panel from Figure 1 and retained it in Figure 2, where direct comparison of PRPF31 protein expression between iPSC-RPE cells and retinal organoids is presented. We have also expanded the Results section to comment on the observed difference in PRPF31 protein upregulation between the two models, noting that PRPF31-AAV treatment resulted in a greater fold-increase in retinal organoids (~3-fold) than in RPE cells (~1.8-fold). (page 7 of revised results: "Notably, the fold-increase in total PRPF31 protein expression was higher in ROs (3-fold) than in transduced RPE cells (1.8-fold). This finding indicates that the PRPF31-AAV vector effectively increased PRPF31 protein levels in both disease-relevant cell types, with a more pronounced response observed in retinal organoids. The higher relative increase in organoids may reflect differences in transduction efficiency, transgene expression, or baseline PRPF31 protein levels between the two cellular models".

3) Lines 177-179, statement "however, a residual cytoplasmic PRPF31 fraction persisted in AAV-transduced cells (23.5%) compared with a negligible cytoplasmic signal in wild-type controls (11). Are the Authors referring exclusively to a previous publication here? The way it is presented seem to indicate results shown in the paper.

In the previous version, we referred to the wild type from our previous publication. However, since we have added wild-type data to Figure 1C, we now compare the PRPF31.AAV directly to the wild-type data within this manuscript. A note has been added to page 6 as follows: The restored localisation pattern closely mirrored that observed in wild-type RPE cells; however, a residual cytoplasmic PRPF31 fraction persisted in AAV-transduced cells (23.5%) compared with the cytoplasmic signal (4.4%) in wild-type controls (**Figure 1C**).

4) Transcriptomic and proteomic analyses: the Authors should provide more details on the number of samples analyzed both for the RNAseq and proteomic analyses. Also, did they use in both cases RP11-RPE and RP11-RO from all the three patients available (mentioned in the Methods)?

We thank the reviewer for this comment. We used RP11S3 RPE cells and RP11S1 ROs for both transcriptomic and proteomic analyses. Biological triplicates from each experimental group were used for both the transcriptomic and proteomic analyses (details added in the legends of Figures 6 and 7).

5) Transcriptomic analysis: The Authors describe a number of differential splicing events between PRPF31.AAV-treated and GFP.AAV-treated samples, both RP11-RPE (241 events) and RP11-RO (261 events) (page 10). It is not clear whether there are alternative splice events that are specific (enriched) in control (GFP)-treated cells vs. PRPF31-treated ones. If so, which are the numbers? A more detailed explanation of these analyses is needed. We thank the reviewer for this comment and present now the significant splicing events enriched in the GFP.AAV (inclusion level difference - positive values) and PRPF31.AAV (inclusion level difference - negative values) treated RPE cells and ROs in the revised Table S2. However, the pathway analysis was focused on PRPF31.AAV-enriched splicing events as we aimed to identify splicing defects rescued by PRPF31 supplementation. A systematic analysis of disease-associated splicing alterations in GFP-treated cells was beyond the scope of this study and has already been addressed in our prior work comparing PRPF31 to wild-type and CRISPR/Cas9 RPE and retinal organoids (Buskin et al., Nat Commun. 2018 Oct 12; 9:4234).

6) Transcriptomic analysis: again, when reporting the results of the enrichment analysis of the genes affected by changes in splicing pattern, the Authors do not mention whether some of these genes also display differential gene expression (Figure 5A-B).
We thank the reviewer for this comment. We have performed this comparison (see Venn diagrams below) but found little overlap between differentially expressed (DE) and differentially spliced (DS) transcripts, corroborating results previously published by our group comparing PRPF31 to CRISPR/Cas9 RPE and ROs (Buskin et al., Nat Commun. 2018 Oct 12; 9:4234). Among the 7 and 8 transcripts common to both the DE and DS sets in RPE cells and ROs, PRPF31 is one of them, consistent with its expected direct response to gene augmentation, and perhaps a feedback loop through autoregulation of its own splicing.

In summary, the limited overlap between differentially expressed and differentially spliced transcripts suggests that PRPF31.AAV primarily restores splicing homeostasis rather than broadly inducing the same transcriptome changes seen at the expression level. This is consistent with previous PRPF31-RPE studies showing that splicing and expression changes only partially intersect, indicating distinct primary and downstream molecular responses (Rodrigues et al. NPJ Regen Med. 2022 Aug 16;7:39; Buskin et al., Nat Commun. 2018 Oct 12; 9:4234). A note to reflect these findings has been added to the revised discussion, page 17.

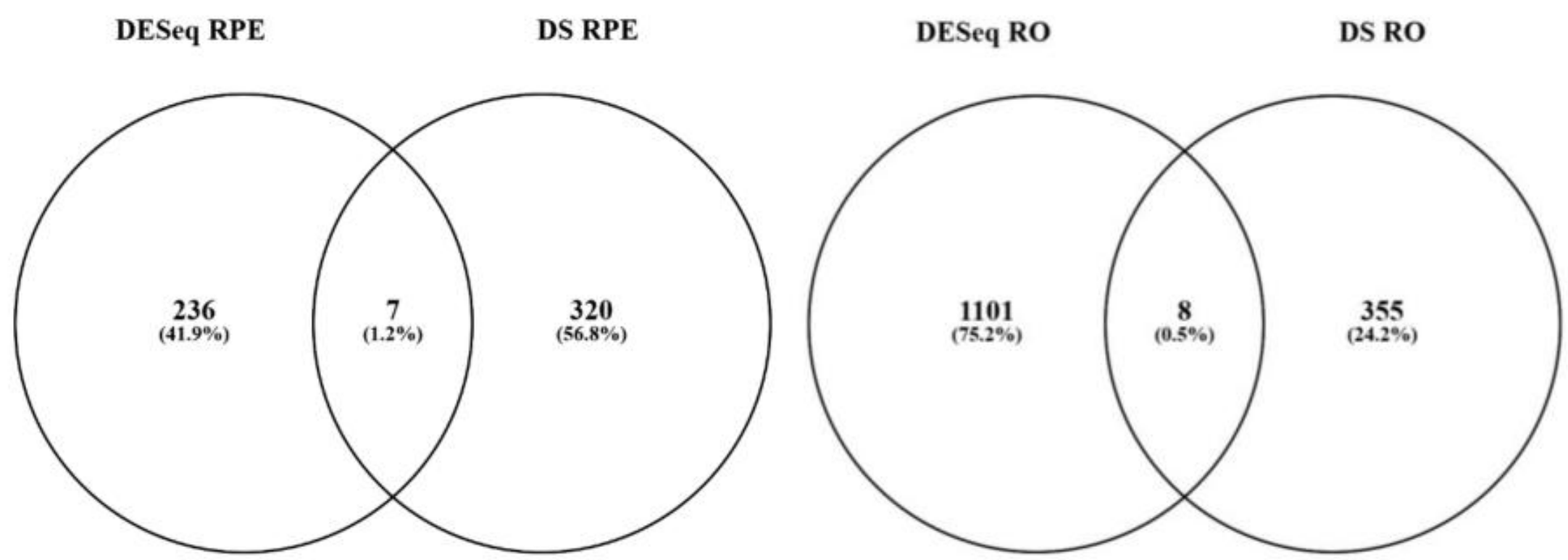

We hope that these revisions are satisfactory. We look forward to hearing from you in due course.

Sincerely (on behalf of all senior authors),

Majlinda Lako, PhD
Professor of Stem Cell Science
